# The association of Emergency Department presentations in pregnancy with hospital admissions for postnatal depression (PND): a cohort study based on linked population data

**DOI:** 10.1186/s12873-017-0123-8

**Published:** 2017-03-23

**Authors:** Fenglian Xu, Elizabeth A. Sullivan, Roberto Forero, Caroline S. E. Homer

**Affiliations:** 1 Faculty of Health, University of Technology Sydney, Ultimo, 2007 Australia; 2 Simpson Centre for Health Service Research, SWS Clinical School and Ingham Institute for Applied Medical Research, University of New South Wales, Liverpool, NSW 2017 Australia

**Keywords:** Emergency Department presentation, Postnatal depression, Pregnancy, Postpartum, Data linkage, Population

## Abstract

**Background:**

To investigate the impact of presenting to an Emergency Department (ED) during pregnancy on postnatal depression (PND) in women in New South Wales (NSW), Australia.

**Method:**

An epidemiological population-based study using linked data from the NSW Emergency Department Data Collection (EDDC), the NSW Perinatal Data Collection (PDC) and the NSW Admitted Patients Data Collection (APDC) was conducted. Women who gave birth to their first child in NSW between 1 January 2006 and 31 December 2010 were followed up from pregnancy to the end of the first year after birth.

**Results:**

The study population includes 154,328 women who gave birth to their first child in NSW between 2006 and 2010. Of these, 31,764 women (20.58%) presented to ED during pregnancy (95%CI = 20.38–20.78). Women who presented to ED during pregnancy were more likely to be admitted to hospital for the diagnosis of unipolar depression (the adjusted relative risk (RR) =1.86, 95%CI = 1.49–2.31) and the diagnosis of mild mental and behavioural disorders associated with the puerperium (the adjusted RR = 1.55, 95%CI = 1.29–1.87) than those without ED presentation.

**Conclusion:**

Women’s hospital admissions for postnatal depression were associated with frequent ED presentations during pregnancy.

## Background

Depression in the postnatal period is a major public health problem that can significantly impact the health of the whole family [1, 2]. One of our previous studies showed that the hospital admission rates for psychiatric disorders in the first year after birth increased significantly between 2001 and 2010, with a more marked increase from 2005 (1.16% in 2001, 2.28% in 2010) [3]. The increase in hospital admissions was mainly attributed to the diagnoses of unipolar depression, adjustment disorders and anxiety disorders [3]. A recent prospective pregnancy cohort study in Melbourne, Australia (*n =* 1507 nulliparous women) showed that 16.1% of women were screened to be at risk of depression (using the Edinburgh Postnatal Depression Scale, EPDS) during the first 12 months postpartum [4]. The study also showed that poor physical health in the early postnatal period was associated with poorer mental health throughout the first 12 months postpartum [4]. For example, women reporting greater than four health problems had a six-fold increase in the likelihood of reporting concurrent depression at three months postpartum (adjusted odds ratio(OR) = 6.69, 95%CI = 3.0–15.0) and a three-fold increase in the likelihood of reporting subsequent depression at 6–12 months postpartum (adjusted OR = 3.43, 95%CI = 2.1–5.5) [4]. Women experiencing a greater number of physical health problems also seem to be at increased risk of reporting depression in early pregnancy [5]. However, the impact of physical health problems during pregnancy on depression after birth (postnatal depression, PND) is unknown. To date, we have not found any literature that explores the association between ED presentation during pregnancy and hospital admission for PND. It is important to understand this association so preventative measures can be put in place if they are required. This study aims to address this gap in this area of research. We have used population-based linked data to:

1. describe the rate of ED presentations for women during pregnancy
2. compare the risks of hospital admission for PND in women with and without ED presentations during pregnancy.

The assumption was that ED presentation in pregnancy is associated with an increased risk of hospital admission for PND. PND refers to the principal diagnoses of depressive disorders including unipolar depressive disorder, mental and behavioural disorders associated with the puerperium and others (the diagnoses are detailed in the methodology section) in the first year after birth.

## Methods

### Study population and design

This is a population-based study using linked data from the NSW Emergency Department Data Collection (EDDC), the NSW Perinatal Data Collection (PDC) and the NSW Admitted Patients Data Collection (APDC). The study included all women who gave birth to their first child in NSW between 1 January 2006 and 31 December 2010. Women who had subsequent births in the two years after the first birth were excluded.

**Fig. 1:**
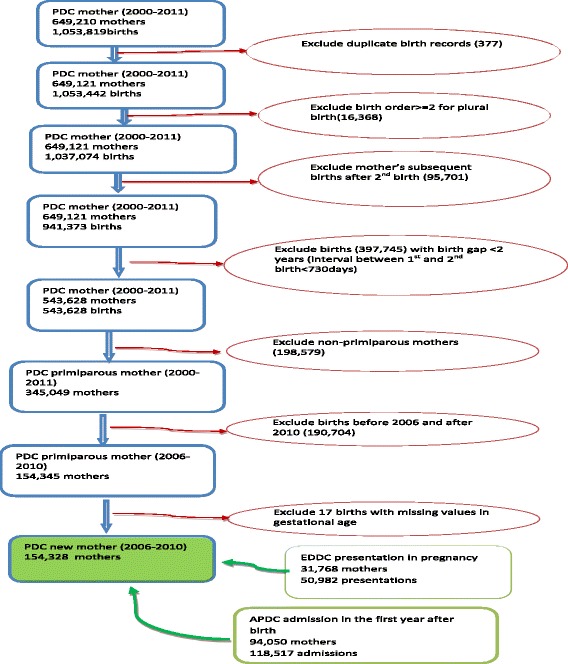
The Data Linkage and Study Population

ED presentations for women during pregnancy were identified by ED records, and hospital admissions for depressive disorders were identified by APDC records. Women’s birth records were linked with the EDDC between 1 January 2005 and 31 December 2010, so that a woman’s ED presentations could be traced back to their pregnancy period. PDC birth records were also linked with APDC records between 1 January 2006 and 31 December 2011, so that hospital admissions for these women could be followed up one year after birth [6]. The study population selection and data linkage are detailed in Fig. 1.

The PDC collects data on all births of at least 20 weeks gestation or at least 400 g birthweight, in NSW, Australia. It covers all births in public hospitals, private hospitals and homebirths in this state and includes information on maternal characteristics, pregnancy, labour, delivery and neonatal outcomes. The APDC is a routinely collected census of all hospital separations. It includes all patient hospitalisations in NSW public and private hospitals, including psychiatric hospitals and day procedures. It includes information on patient demographics, diagnoses and clinical procedures. Since 1999, the diagnoses for admissions have been coded according to the 10th revision of the International Statistical Classification of Diseases and Related Health Problems, Australian Modification (ICD-10-AM) [7]. The EDDC is the primary data source from the EDs of NSW public hospitals. It contains patient demographics (e.g. age, country of birth, sex and marital status), time-tracking information, including arrival and discharge dates, and triage category, mode of admission, disposition and the diagnosis.

Data linkage was performed by the NSW Department of Health Centre for Health Record Linkage (CHeReL) using probabilistic record linkage methods and Choicemaker software [8]. Identifying information from PDC, EDDC and APDC datasets was included in the Master Linkage Key constructed by the CHeReL. At the completion of the process, each record was assigned a Person Project Number to allow records for the same individual to be linked. Based on the 1,000 randomly selected sample of records, the false positive rate of the linkage was 0.3% and false negative <0.5%.

### Definitions

The principal diagnosis was the diagnosis which was chiefly responsible for the APDC hospital admission [9]. The emergency presentation included any attendance of patient for an actual or suspected condition to ED. Hospital admission referred to a patient who was admitted to hospital for inpatient service. Postnatal depression (PND) referred to the principal diagnoses of depressive disorders including unipolar depressive disorder, mental and behavioural disorders associated with the puerperium and others in the first year after birth, including the day of birth. (the codes of the diagnoses are listed in the paragraph below)

### Diagnosis of depressive disorders

The diagnoses for each admission in this study have been coded according to ICD-10-AM [7]. Women with depressive disorders were identified using ICD-10-AM diagnosis codes: (1) F32 [unipolar depressive disorder]; (2) F53 [mental and behavioural disorders associated with the puerperium] including F53.0 [mild mental and behavioural disorders associated with the puerperium, not elsewhere classified]; F53.1 [severe mental and behavioural disorders associated with the puerperium, not elsewhere classified]; F53.8 [other mental and behavioural disorders associated with the puerperium, not elsewhere classified] and F53.9 [Puerperal mental disorder, unspecified]; (3) Others including F33 [recurrent depressive disorder excluding the in remission three-digit code F33.4]; F34.1 [dysthymia]; F38 [other specified affective disorder]; and F39 [unspecified mood (affective) disorder].

In this study, only the hospital admission with a *principal* diagnosis in the first year after birth was used for the analysis of the rate, relative risk (RR) and adjusted RR.

### Statistical analysis

Descriptive statistics were used to analyse the rate of hospital admission. Logistic regression was used to estimate the adjusted odds ratio (AOR) of women’s characteristics on ED presentation. Cox regression was used to estimate the RR of ED presentations during pregnancy on hospitalisation for PND. The RR was adjusted (ARR) for maternal age, maternal country of birth, maternal diabetes mellitus and hypertension, gestational diabetes, smoking status during pregnancy, remoteness of living area, a socio-economic indicator (i.e. the Index of Relative Socio-economic Disadvantage Quintile) [10], mode of birth, infant gender and birthweight. There were missing values in 3,414 women’s records (2.21%). The variables with missing values included remoteness of living area (2585, 1.68%), a socio-economic indicator disadvantage (2585, 1.68%), smoking status during pregnancy (565, 0.37%), mode of birth (108, 0.07%), infant birthweight (99, 0.06%), baby’s gender (60, 0.04%) and maternal age (31, 0.02%). There were no missing values in variable of maternal country of birth, maternal and gestational diabetes mellitus, and maternal hypertension. The analyses were conducted using IBM SPSS (Statistical Package for the Social Sciences) Statistics 22) [11].

### Ethics approval

This study was approved by the NSW Population and Health Services Research Ethics Committee and the Human Research Ethics Committee of the University of New South Wales and University of Technology Sydney, Australia (reference number: 2011/06/328).

## Results

The study population comprised primiparous women who gave birth in NSW between 2006 and 2010. There were 154,328 women who met the criteria and were included in the data analysis. Women’s characteristics, demographic factors and ED presentations are described in Table 1. A total of 31,764 women (20.58%) presented to ED during pregnancy in NSW between 2005 and 2010 (95%CI = 20.38–20.78). The ED presentation during pregnancy was associated with maternal age. ED presentation decreased significantly as age increased for women under 40 years old. Women aged between 35 and 39 years had the lowest ED presentation rate (rate = 13.50%, 95%CI = 13.03-13.97; AOR = 0.72, 95%CI = 0.74–0.80 compared with women aged 25 and 29). Young women were more likely to visit the ED (rate = 39.07%, 95%CI = 38.14-40.00 for women aged below 20; AOR = 2.11, 95%CI = 2.02–2.22 compared with those aged between 25 and 29 years). ED presentation was significantly associated with relative socio-economic disadvantage, smoking in pregnancy, maternal and gestational diabetes mellitus, and maternal hypertension. Women born outside Australia were less likely to visit the ED during pregnancy (Table 1). Compared with women living in major cities, those living in inner regional areas were more likely to visit ED but those living in outer regional and remote areas were less likely to visit ED during pregnancy (Table 1).

**Table 1:** Primiparous women’s characteristic and ED presentation in pregnancy in NSW, 2005–2010

Indicator	Value	Woman	ED presentations in pregnancy	Rate	AOR	95%CI	
Maternal age*	<20	10601	4142	39.07	2.11	2.02	2.22
	20–24	27718	8693	31.36	1.66	1.60	1.72
	25–29	46949	9095	19.37	1.00		
	30–34	44489	6439	14.47	0.77	0.74	0.80
	35–39	20117	2715	13.50	0.72	0.68	0.75
	40–44	4150	635	15.30	0.82	0.75	0.90
	45+	273	40	14.65	0.76	0.54	1.07
	Total	154297^a^	31759	20.58			
Women’s country of birth*	Australia	101122	22805	22.55	1.00		
	Others countries	53206	8959	16.84	0.87	0.84	0.90
	Total	154328	31764	20.58			
Remoteness*	Major cities	108118	19770	18.29	1.00		
	Inner regional	32862	9061	27.57	1.31	1.26	1.35
	Out regional and remote	10763	2549	23.68	0.80	0.76	0.84
	Total	151743^b^	31380	20.68			
Smoking during pregnancy*	No	138800	26526	19.11	1.00		
	Yes	14963	5149	34.41	1.49	1.43	1.55
	Total	153763^c^	31675	20.60			
Index of Relative SE Disadvantage Quintile*	Least disadvantaged	36071	4571	12.67	1.00		
	2	31597	5846	18.50	1.31	1.26	1.37
	3	28958	6432	22.21	1.44	1.38	1.50
	4	26358	6823	25.89	1.58	1.51	1.65
	Most disadvantaged	28759	7708	26.80	1.83	1.75	1.91
	Total	151743^d^	31380	20.68			
Maternal diabetes mellitus*	No	153510	31490	20.51	1.00		
	Yes	818	274	33.50	2.02	1.74	2.35
	Total	154328	31764	20.58			
Gestational diabetes*	No	146549	30167	20.58	1.00		
	Yes	7779	1597	20.53	1.19	1.12	1.26
	Total	154328	31764	20.58			
Maternal hypertension*	No	153091	31475	20.56	1.00		
	Yes	1237	289	23.36	1.22	1.07	1.40
	Total	154328	31764	20.58			

*ED* Emergency Department, *SE* socio-economic, *AOR* adjusted odds ratio. The adjusted factors include all indicators in Table 1

*Significantly associated with ED presentation during pregnancy by logistic regression analysis (*p <* .05)

^a^31 missing data, ^b^2585 missing data, ^c^565 missing data, ^d^2585 missing data, see Methods

Overall, 21,357 visits (41.89%) were made by women presenting to the ED only once whereas 10,407 women made multiple presentations, accounting for 29,625 (58.11%) of the 50,982 total number of presentations during the observation period). Of the 31,764 women who presented to the ED during the 10-month observation period, 20.58% (31,764/154,328) made at least one visit and 12.45% (19,218/154,328) made at least one subsequent visit. The total ED presentation rate was 33.03% (50,982/154,328, 95%CI = 32.80–33.26). Using person-year as the denominator for comparison with another period or another population, the first presentation during the observation period was 27.40/100 person-year (31,764/115,911, 95%CI = 27.14–27.66/100); the re-presentation during the observation period was 16.58/100 person-year (19,218/115,911, 95%CI = 16.37–16.79/100). The total presentation during the observation period was 43.98/100 person-year) (50,982/115,911, 95%CI = 43.69–44.27/100).

**Fig. 2:**
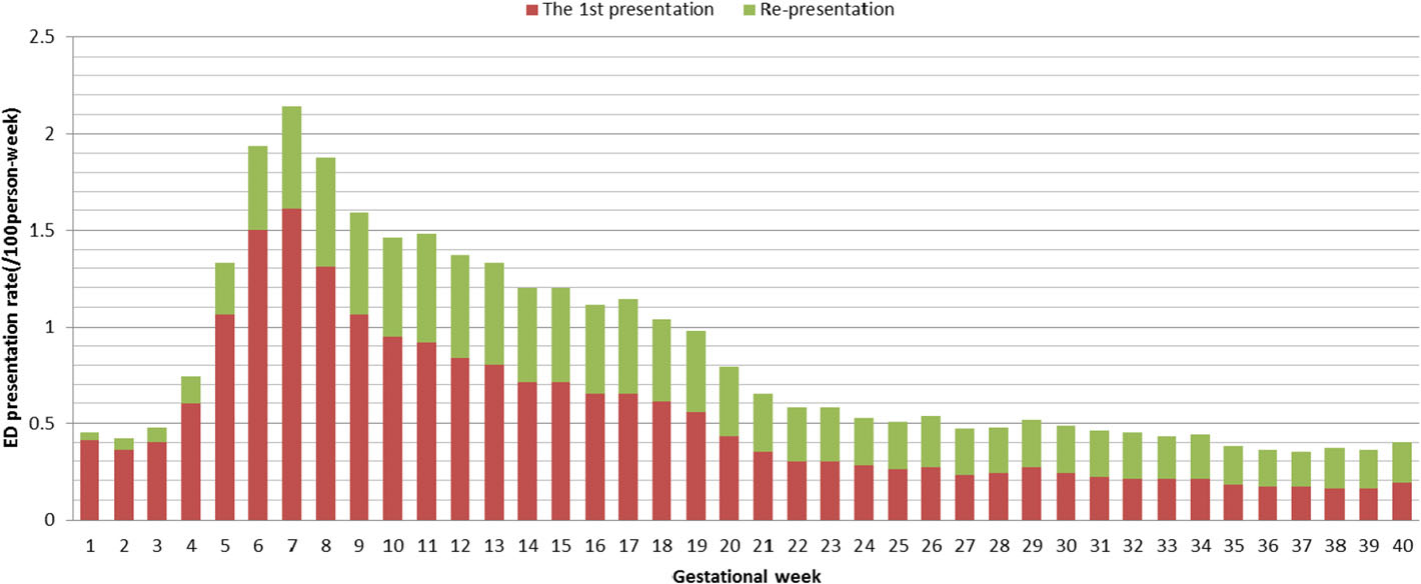
The Rate of Emergency Department Presentations During Pregnancy, New South Wales, 2005–2010

Figure 2 shows the distribution of ED presentations and the rate of ED presentation over gestational weeks. The ED presentations were below 0.50/100 person-week in the first 3 gestational weeks. Since the 4^th^ gestational week, the ED presentations increased significantly and peaked in the 7th gestational week (2.14/100 person-week, 95%CI = 1.91–2.37) and then declined significantly until 22 weeks (Fig. 2).

**Fig. 3:**
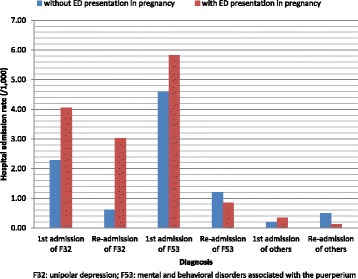
The Hospital Admission Rate (/1,000) of Women with and without Emergency Department Presentation During Pregnancy, New South Wales, 2005–2010

Table 2 shows the hospital admissions for PND in women with and without ED presentations during pregnancy. Women who presented to ED during pregnancy were more likely to be admitted to hospital for the diagnosis of unipolar depression (F32) (rate = 7.08/1000, 95%CI = 6.16–8.00) than those without an ED presentation (rate = 2.89/1000, 95%CI = 2.59–3.19). There was no significant difference in hospital admission rates for the diagnosis of mild or severe mental and behavioural disorders associated with the puerperium (F53.0 and F53.1) between women with and without ED presentations during pregnancy (Table 2 and Fig. 3).

**Table 2:** Hospital admissions for postnatal depression in women with and without ED presentations during pregnancy, NSW 2006–2011

Principal diagnosis	ED presentation during pregnancy	Woman	The 1st admission				Re-admission				Overall admission			
			Admission	Rate (/1,000)	95% CI		Admission	Rate (/1,000)	95% CI		Admission	Rate (/1,000)	95% CI	
Unipolar depressive disorder (F32)	No	122564	279	2.28	2.01	2.55	75	0.61	0.47	0.75	354	2.89	2.59	3.19
	Yes	31764	129	4.06	3.36	4.76	96	3.02	2.42	3.62	225	7.08	6.16	8.00
	Total	154328	408	2.64	2.38	2.90	171	1.11	0.94	1.28	579	3.75	3.45	4.05
Mild MBDP (F53.0)	No	122564	479	3.91	3.56	4.26	63	0.51	0.38	0.64	542	4.42	4.05	4.79
	Yes	31764	158	4.97	4.20	5.74	15	0.47	0.23	0.71	173	5.45	4.64	6.26
	Total	154328	637	4.13	3.81	4.45	78	0.51	0.40	0.62	715	4.63	4.29	4.97
Severe MBDP (F53.1)	No	122564	97	0.79	0.63	0.95	64	0.52	0.39	0.65	161	1.31	1.11	1.51
	Yes	31764	25	0.79	0.48	1.10	10	0.31	0.12	0.50	35	1.10	0.74	1.46
	Total	154328	122	0.79	0.65	0.93	74	0.48	0.37	0.59	196	1.27	1.09	1.45
MBDP (F53)	No	122564	562	4.59	4.21	4.97	147	1.20	1.01	1.39	709	5.78	5.36	6.20
	Yes	31764	185	5.82	4.98	6.66	27	0.85	0.53	1.17	212	6.67	5.77	7.57
	Total	154328	747	4.84	4.49	5.19	174	1.13	0.96	1.30	921	5.97	5.59	6.35
Others	No	122564	23	0.19	0.11	0.27	61	0.50	0.37	0.63	84	0.69	0.54	0.84
	Yes	31764	11	0.35	0.17	0.63	4	0.13	0.04	0.33	15	0.47	0.26	0.78
	Total	154328	34	0.22	0.15	0.29	65	0.42	0.32	0.52	99	0.64	0.51	0.77
Overall depressive disorders	No	122564	815	6.65	6.19	7.11	332	2.71	2.42	3.00	1147	9.36	8.82	9.90
	Yes	31764	304	9.57	8.50	10.64	148	4.66	3.91	5.41	452	14.23	12.93	15.53
	Total	154328	1119	7.25	6.83	7.67	480	3.11	2.83	3.39	1599	10.36	9.85	10.87

*ED* Emergency Department, *MBDP* Mental and behavioural disorders associated with the puerperium (F53)

Hospital readmissions were not homogeneously distributed. The readmissions were attributed mainly to a small number of women.

For the sub-categories of F53, F53.8 (other mental and behavioural disorders associated with the puerperium, not elsewhere classified) and F53.9 (puerperal mental disorder, unspecified), the numbers of hospital admissions were too small to calculate their specific rates and RRs (8 hospital admissions with the diagnosis of F53.8 and 2 admissions with the diagnosis of F53.9). However, the admissions of diagnoses were included in the analysis of overall F53.

Table 3 shows that the risk of hospital admission for PND was higher in women who presented to ED during pregnancy than without ED presentation (adjusted RR = 1.64, 95%CI = 1.43–1.88). The adjusted RR was 1.86 (95%CI = 1.49–2.31) for the diagnoses of unipolar depressive disorder (F32) and 1.55 (95%CI = 1.29–1.87) for mild mental and behavioural disorders associated with the puerperium (F53.0). The adjusted factors include maternal age, maternal country of birth, maternal diabetes mellitus and hypertension, gestational diabetes, smoking status during pregnancy, remoteness of living area, Index of Relative Socio-economic Disadvantage Quintile, mode of birth, infant gender and birthweight. The adjusted RR for severe mental and behavioural disorders associated with the puerperium (F53.1) was not statistically significant. The adjusted RR for other depressive disorders was 1.90, but the difference was not statistically significant (95%CI = 0.90–4.00). This may be attributed to the small number of women (only 34 women were admitted to hospital for this diagnosis).

**Table 3:** The relative risk (RR) of hospital admissions for postnatal depression in women with and without ED presentations during pregnancy, NSW 2006–2011

Principal diagnosis	ED presentation during pregnancy	Woman	RR	95% CI		Adjusted RR^a^	95% CI	
Unipolar depressive disorder (F32)	No	122564	1			1		
	Yes	31764	1.79	1.45	2.20	1.86	1.49	2.31
Mild MBDP (F53.0)	No	122564	1			1		
	Yes	31764	1.27	1.06	1.52	1.55	1.29	1.87
Severe MBDP (F53.1)	No	122564	1			1		
	Yes	31764	0.99	0.64	1.54	1.17	0.74	1.85
MBDP (F53)	No	122564	1			1		
	Yes	31764	1.27	1.08	1.50	1.53	1.28	1.81
Others	No	122564	1			1		
	Yes	31764	1.85	0.90	3.79	1.90	0.90	4.00
Overall depressive disorders	No	122564	1			1		
	Yes	31764	1.44	1.26	1.64	1.64	1.43	1.88

*ED* Emergency Department *RR* relative risk, *MBDP* Mental and behavioural disorders associated with the puerperium (F53)

^a^Adjusted for maternal age, maternal country of birth, maternal diabetes mellitus and hypertension, gestational diabetes, smoking status during pregnancy, remoteness of living area, Index of Relative, Socio-economic Disadvantage Quintile, delivery method, infant gender and birthweight

## Discussion

**Fig. 4:**
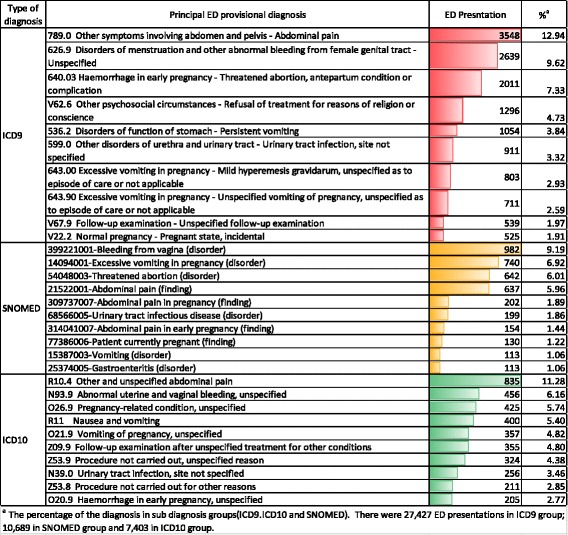
The Top 10 Frequent Diagnoses for Women’s Emergency Department Presentation

The association between ED presentation and PND is not well investigated [12, 13]. This study has shown that women who visited the ED during pregnancy were more like to be admitted to hospital for PND after birth. This leads on from a prospective study in six metropolitan public maternity hospitals in Victoria, Australia which showed that women reporting five or more physical health problems (e.g. exhaustion, morning sickness, back pain, constipation and severe headaches or migraines) in early pregnancy had a three-fold increase of depressive disorders (adjusted OR = 3.13, 95%CI = 2.14–4.58) in early pregnancy (gestation *=* 15 weeks) [5]. A study in 181 women showed that women’s distress in the week following childbirth was associated with more frequent vomiting during the first 3–4 months of pregnancy [14]. The most common reasons for women visiting ED during pregnancy were haemorrhage and bleeding, abdominal pain and vomiting (Fig. 4). The most common gestation was seven weeks suggesting that early pregnancy issues were the main reason for ED presentation.

The ED presentation rate in pregnancy from this data was 43.98/100 person-year (95%CI = 43.69–44.27/100 person-year). The rate was significantly higher than for the general population in NSW and Australia [15]. Based on the hospital statistics and population reports by the Australian Bureau of Statistics, omit ED presentation rates in NSW were estimated at 28.47% (person-year) (2,007,864/7,053,755) in 2008–2009 [1, 13, 16]. The ED presentation rate in Australia was estimated at 26.47% (person-year) (5,742,139/21,691,653) in 2008–2009 [15, 16]. Australian hospital statistics for 2012–2013 show that there were more than 6.7 million ED presentations for the period July 2012 to June 2013 [15]. The ED presentation rate in Australia was lower than for the United States. An overview of ED visits in the United States showed that there were approximately 421 visits to the ED for every 1,000 individuals in the population in 2011, and women visited an ED 20% more often than men (458 versus 382 per 1,000 population) [17].

In many countries, primary health care providers are experiencing an increased demand for Emergency Department (ED) services [15, 17]. In Australia, some studies have reported increases in estimates of between 2% and 4% in ED presentations in New South Wales (NSW) (2001–2011) [16], Western Australia (WA) (2006–2013) [18] and Victoria (1999–2009) [19]. A recent study from WA has predicted a 4.8% increase per annum in ED presentations in the next five years [18]. However, there is little literature on the ED presentation rates because of a lack of accurate population data to use as the denominator for the rate analysis. Data from women who have given birth provide a reliable means to calculate the ED presentation rate in women who have given birth.

Our study also shows that the hospital admission rate for depression in the first year after birth in women was 1.04% (95%CI = 0.99–1.09%). The rate was higher than in a previous study (0.49%) in NSW (2002–2004) [20]. The rate was also higher than in other countries, for example, Denmark [21]. A Danish population-based cohort study (1973–2005) showed that the rate of first-time hospital admissions for mental disorders was 0.6% in the first month after birth [21]. The variation may be attributed to differences in study year and health service accessibility. Our previous study showed that hospital admission rates for psychiatric disorders in the first year after birth increased significantly between 2001 and 2010, with a more marked increase from 2005 (1.16% in 2001, 2.28% in 2010) [8].

The strengths of this study are the size of the study population and consecutive follow up of ED presentations and hospital admissions for depression in the first postpartum year. The large population size (over 154,000) women who gave birth for the first time, and who were followed up from pregnancy until the end of the first year after birth, allowed us to examine the distributions of ED presentations over the period of pregnancy and hospital admissions for depression over the 1^st^ year after birth.

## Limitations

It is possible that the hospital admission for PND may be over-enumerated, and thus the rate of PND overestimated, because admission could occur for medical reasons associated with the postnatal period [22, 23]. To minimise the potential over-estimation of incidence rates, we included only those admissions with a ‘principal’ diagnosis of depressive disorder (i.e. the diagnosis primarily responsible for the admission).

Unlike the NSW APDC, which has diagnoses coded by trained clinical information managers, the EDDC has substantial limitations in coding of diagnoses. The diagnoses in EDDC were recorded by medical, nursing or clerical personnel at the point of care. These personnel were not trained in clinical coding and the diagnoses were selected by keyword searching or tables of a limited set of diagnoses. The codes were assigned to the chosen diagnosis using tables built into the computer database program. There are several different computer programs used in EDs. These programs use different classifications to record the diagnosis, including ICD-9-CM (Clinical Modification), ICD-10-AM (Australian Modification) or SNOMED CT (the Systematized Nomenclature of Medicine – Clinical Terms) [24]. It is difficult to map and group the diseases or symptoms because of the variation in computer programs and completeness of diagnoses. For example, some disease categories are not available in some programs, but may be in others. A code commonly recorded (2.85% of presentations with an ICD-10 diagnosis) was ‘Z53.8 – procedures not carried out for other reasons’. This may reflect workflow processes or the inappropriateness of the use of ICD coding in this context.

The diagnoses for ED presentation were incomplete. The records of only 89.28% of the ED presentations (45,519/50,982) included the diagnoses (ICD-9 or ICD-10 or SNOMED). Of the ED presentations that included the diagnoses, 60.25% (27,427) presentations had ICD-9 diagnoses; 23.48% (10,689) had SNOMED diagnoses and 16.26% (7,403) had ICD-10 diagnoses. The top 10 diagnoses for ICD-9, SNOMED and ICD-10 respectively are described in Fig. 4.

Some predisposing factors for PND such as family support and income were not available in the data. A contributing factor could have been a history of mental health problems, however this was not assessed here.

## Conclusion

The presentation of women to an ED during pregnancy was associated with a higher risk of admission to hospital with a diagnosis of postnatal depression. The reasons for this need to be explored further in a future study. Clinicians should consider the association between ED admission and a diagnosis of postnatal depression when planning a woman’s care.

## Abbreviations

APDC: Admitted Patients Data Collection
CHeReL: Centre for Health Record Linkage
CI: confidence interval
ED: Emergency Department
EDDC: Emergency Department Data Collection
ICD-10-AM: International Classification of Diseases and Related Health Problems 10th Revision Australian Modification
ICD-9-CM: International Classification of Diseases and Related Health Problems, 9th Revision, Clinical Modification
NHMRC: National Health and Medical Research Councill
NSW: New South Wales
OR: Odds ratio
PDC: Perinatal Data Collection
PDD: Postnatal depressive disorders
PND: Postnatal depression
RR: Relative risk
SNOMED CT: Systematized Nomenclature of Medicine – Clinical Terms
